# Encyclopædia Americana: Supplementary Volume

**Published:** 1847-05

**Authors:** 


### BIBLIOGRAPHICAL NOTICES.

---

ART. V.—*Encyclopædia Americana: Supplementary Volume. A Popular Dictionary of Arts, Sciences, Literature, History, Politics and Biography.* Vol. XIV. Edited by HENRY VETHAKE, LL.D., Vice-Provost and Professor of Mathematics in the University of Pennsylvania; Member of the American Philosophical Society; Author of a Treatise on Political Economy, etc. 8vo. pp. 663. Philadelphia: Lea and Blanchard: 1847.

The Encyclopœdia Americana, published a number of years since, is so well known and so highly approved, that no higher recommendation of the supplementary volume is needed than to say, that it is worthy of a place by the side of its compeers. Every one who possesses the original work will be desirous of procuring the supplement, in which he will find a large and varied fund of useful information on biography, politics, history, literature and science.

